# Reading the signs: Camera‐trapping provides new insights on scent marking in the large‐antlered muntjac (*Muntiacus vuquangensis*)

**DOI:** 10.1002/ece3.9692

**Published:** 2023-01-16

**Authors:** Andrew Tilker, Pablo Sinovas

**Affiliations:** ^1^ Re:wild Austin Texas USA; ^2^ Leibniz Institute for Zoo and Wildlife Research Berlin Germany; ^3^ Fauna & Flora International Phnom Penh Cambodia

**Keywords:** Annamites, camera‐trap, intraspecific communication, large‐antlered muntjac, scent marking, Southeast Asia

## Abstract

We present evidence of scent marking in the large‐antlered muntjac (*Muntiacus vuquangensis*). Given the importance of scent marking in individual recognition among ungulates, this behavior may serve to communicate the fitness cost of antagonistic interactions among rival males and could serve as a mechanism for mate assessment among females.

## Graphical abstract

We document the first recorded evidence of scent marking in the large‐antlered muntjac (*Muntiacus vuquangensis*), a little‐known and threatened deer endemic to the Annamites ecoregion of Indochina. We discuss the function of this behavior within a wider behavioral and evolutionary ecology framework for mammals living in tropical forests.

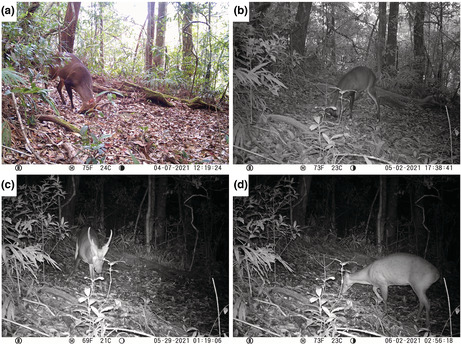

Indirect forms of communication play an important role in the exchange of information among mammals. Scent marking, in particular, appears to be widespread among a range of species (Gosling & McKay, 1990; Johnson, 1973; MacDonald, 1980). Scent marking has advantages over other forms of indirect communication because olfactory signals can persist in an environment long after an animal has left an area, thus increasing the spatial and temporal scope of information transfer (Hurst, 2005). Such considerations may be particularly important where direct interactions are infrequent; for example, in species with solitary social structures (Allen et al., 2016; Hou et al., 2021), or in dense habitats where auditory or visual cues have limited range (Bowyer et al., 1994; Filipczyková et al., 2017; Wegge & Mosand, 2015).

There are other advantages to using scent marking as a form of communication: For many species, chemicals in scent signals can encode information about individual identity and physical characteristics (Brennan & Kendrick, 2006; Gosling, 1982; Gosling & Roberts, 2001). Scent marking can therefore be used as a sophisticated way to broadcast information to conspecifics, and may be used to define territories (Brashares & Arcese, 1999a, 1999b; Rajagopal et al., 2011), advertise presence to potential competitors (Johansson & Liberg, 1996), communicate social status (Hurst et al., 2005; Ralls, 1974), or to attract potential mates (Deutsch & Nefdt, 1992; Rich & Hurst, 1998).

Because scent marking is so prominent among mammals, understanding its role in indirect communications is crucial for obtaining a holistic picture of mammalian behavioral ecology. Here, we report observations of scent marking by the large‐antlered muntjac (*Muntiacus vuquangensis*), a solitary and elusive deer found in the tropical forests of Laos, Viet Nam, and Cambodia (Timmins et al., 1998). The large‐antlered muntjac is also one of the most threatened deer species in Southeast Asia; its populations have collapsed as a result of intensive and unsustainable hunting across its range, and it is listed as Critically Endangered on the IUCN Red List of Threatened Species (Timmins et al., 2016). The observations are, to our knowledge, the first time scent marking has been discussed for large‐antlered muntjac, and provide novel insights into the behavior of one of Southeast Asia's most secretive ungulates.

From 22 February 2021 to 27 July 2021, we deployed camera‐traps in Virachey National Park, in northeastern Cambodia, as part of a biodiversity survey to document the terrestrial wildlife in the protected area. From 7 April to 24 June, one camera‐trap (Bushnell HD Trophy Cam) documented 11 independent encounters (independence threshold = 60 min) of large‐antlered muntjac in closed‐canopy tropical forest habitat. Large‐antlered muntjac were distinguished from the morphologically similar and sympatric northern red muntjac (*Muntiacus vaginalis*) based on antler configuration in males, forehead coloration in females, and length of the tail in both sexes (Alexiou et al., 2022; Schaller, 1996; Timmins et al., 1998).

The camera captured three sequences of a male muntjac vigorously rubbing its head on the ground, presumably to deposit scent using its frontal or forehead glands, which are well‐developed in muntjac (Barrette, 1975). The muntjac also scraped the ground with its front hooves and, on one occasion, urinated at the marking (Figure 1; Table 1). Based on antler configuration, these sequences represent one, and potentially two, individuals. In all sequences, the muntjac also explored the surrounding area by sniffing the ground and vegetation. The average (mean ± standard error) time spent at the site for the marking muntjac was 12:52 ± 10:99 min; in the third sequence alone, the male spent 34:50 min at the location. There were four more sequences of a single male muntjac, identified by distinctive light‐colored and thin antlers, passing through the area and sniffing the ground but not marking. This nonmarking male spent an average of 1:15 ± 0:65 min at the site. Finally, there were four sequences of a single female muntjac, identified by a tear in the right ear, investigating the area by sniffing the ground and vegetation. The female spent an average of 1.04 ± 0.64 min at the location.

**FIGURE 1:**
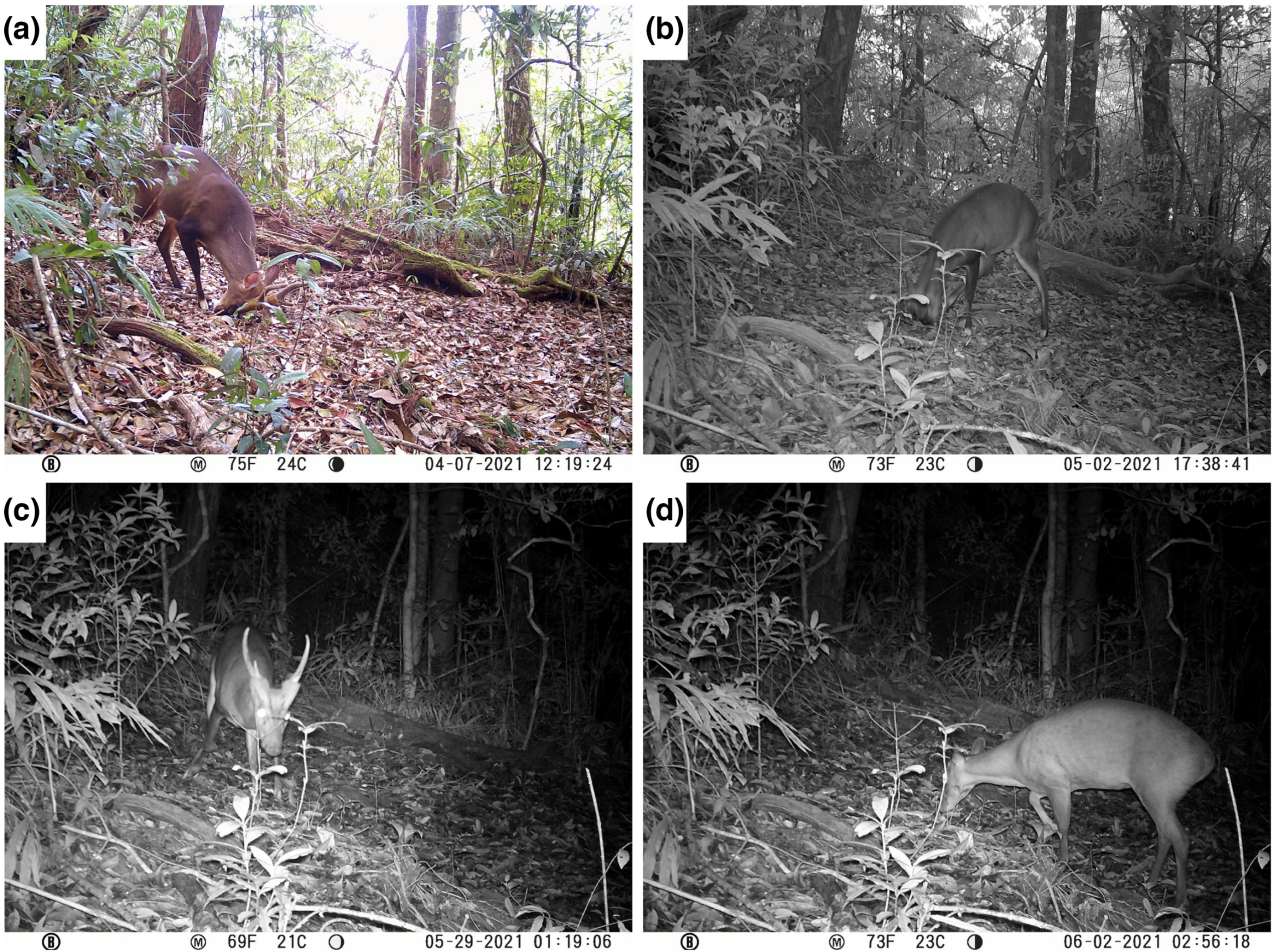
Large‐antlered muntjac (*Muntiacus vuquangensis*) marking and investigative behavior captured by camera‐trap in Virachey National Park, Cambodia. (a) First occasion of male scent marking by rubbing forehead on the ground; (b) second occasion of male scent marking by rubbing forehead on the ground; (c) different nonmarking male investigating scent marked area; and (d) female investigating scent marked area

**TABLE 1:** Timeline of camera‐trap documented activities of large‐antlered muntjac (*Muntiacus vuquangensis*) scent marking and investigative behavior

Time beginning	Time end	Activity description
12:19:24	12:21:16	Male muntjac marking the ground with forehead, then sniffing surrounding area (Figure 1a)
17:37:52	17:39:47	Male muntjac marking the ground with forehead, then sniffing surrounding area, potentially the same individual as 7 Apr 2021 (Figure 1b)
1:19:00	1:20:00	Male muntjac investigates the area, sniffing the ground; different individuals from 7 Apr 2021 or 2 May 2021 (Figure 1c)
2:52:37	2:56:22	Female muntjac investigative behavior, sniffing the ground and exploring surroundings (Figure 1d)
0:25:56	0:26:12	Same female muntjac shows investigative behavior, sniffing the ground and exploring surroundings
17:18:41	17:18:51	Same female muntjac shows investigative behavior, sniffing the ground and exploring surroundings
16:40:23	16:40:41	Same female muntjac shows investigative behavior, sniffing the ground and exploring surroundings
12:45:12	12:46:03	Same female muntjac shows investigative behavior, sniffing the ground and exploring surroundings
12:55:12	12:55:23	Male muntjac investigates the area, sniffing the ground; same individual from 29 May 2021
16:19:34	16:54:24	Male muntjac marking the ground with forehead, urinating, then sniffing surrounding area; potentially the same individual as 7 Apr 2021 or 2 May 2021
23:24:41	23:27:16	Male muntjac investigates the area, sniffing the ground; same individual as 29 May 2021 and 19 Jun 2021

This is, to our knowledge, the first time that scent‐marking behavior has been documented for large‐antlered muntjac. In this way, these findings add to a growing number of recent studies that are beginning to shed light on the species' natural history (Alexiou et al., 2022; Nguyen et al., 2022). Interpreting this behavior is complicated by the fact that these sequences provide a limited window into what are likely complex intraspecific interactions that play out over spatial scales larger than that which is captured by a single camera‐trap; nonetheless, other observations on mammalian scent marking (Estes, 2012), and more specifically on the behavior of muntjac (Barrette, 1975), provide some clues.

Early hypotheses to explain marking behavior focused on marking as a way to delineate and defend territories (Hediger, 1949). Later hypotheses built upon this theme but suggested that the role of marking in intraspecific communication can also have more complex functions. Ralls (1971) argued that mammals engage in scent marking to express dominance to subordinates and that this behavior can, but does not necessarily need to be, linked to territoriality. Gosling (1982) further expanded on this dominant‐subordinate theme, contending that scent marking allows individuals to assess the social status of conspecifics. In an evolutionary context, scent marking may thus be a way for dominant individuals to signal fitness costs to receivers, and could serve as a form of competitive assessment that influences future intra‐species interactions (Gosling & Roberts, 2001; Hurst et al., 2005; Rich & Hurst, 1998).

At the most basic level, it is likely that male large‐antlered muntjac use scent marking to help define territories. However, following Gosling's (1982) hypothesis, we also believe that it is plausible that large‐antlered muntjac use scent marking as a way to advertise social status to potential rivals, and in doing so, assess the fitness cost of potentially antagonistic encounters with conspecifics. Similar behaviors have been observed for other ungulates, including gazelles *Gazella* spp. (Blank et al., 2014; Estes, 2012), musk deer *Moschus chrysogaster* (Green, 1987), water deer *Hydropotes inermis* (Sun et al., 1994), and roe deer *Capreolus capreolus* (Carranza & Mateos‐Quesada, 2001; Johansson et al., 1995). Although no studies have been done on large‐antlered muntjac social behavior, studies on closely related muntjac species indicate that males have well‐defined home ranges with minimal overlap and high site fidelity, with male home ranges overlapping with multiple female home ranges (Chapman et al., 1993; McCullough et al., 2000; Odden & Wegge, 2007). Because males defend their home ranges through sometimes violent interactions (Barrette, 1975), there would be strong evolutionary pressure for males to use scent marking to gauge the relative cost or benefit of challenging other males and thus minimize unnecessary competitive engagements.

In this scenario, the marking muntjac would be the resident male, and the scent marking would function as a way to communicate the cost of antagonistic interactions to potential intruders, thus avoiding an escalation that could result in injury or the high energetic costs often associated with combat. The nonmarking male in this example would represent an intruder, perhaps a dispersing subadult in the process of seeking to establish its own territory.

An alternative explanation is needed to explain the behavior of the female muntjac. In territorial systems, females may increase their fitness by mating with males that are successful at occupying and holding areas. In such an arrangement, matching the scent of a potential mate with the predominant scent markings in an area would provide a way for a female to make such an assessment (Gosling, 1982). Another intriguing possibility is that olfactory cues in male scent deposits could be associated with the synchronization of estrus in females, as has been reported in other deer species (Sawyer et al., 1989). However, without chemical analyses of the pheromones deposited in male muntjac scent markings, this remains speculative.

Scent marking is an important form of indirect communication among mammals but remains little explored for many species. To our knowledge, this is the first time that scent marking has been described for large‐antlered muntjac. By documenting this observation, we seek to lay a foundation for future studies to investigate the role of scent marking in large‐antlered muntjac behavior, and indeed, for other ungulates living in Southeast Asian tropical forests. Given the increase in camera‐trapping in Southeast Asia in recent years, it is likely that additional scent‐marking behaviors will be captured for large‐antlered muntjac and other ungulate species in the region. We would expect that observations over larger spatial areas will allow for more in‐depth inferences to be made about the role of scent marking in indirect communication among Southeast Asian ungulate species. Even more ambitious studies, involving telemetry or the chemical analysis of scent deposits, would help further clarify this aspect of ungulate behavior.

## AUTHOR CONTRIBUTIONS

**Andrew Tilker:** Conceptualization (lead); formal analysis (lead); methodology (lead); writing – original draft (lead); writing – review and editing (equal). **Pablo Sinovas:** Conceptualization (supporting); funding acquisition (lead); methodology (supporting); project administration (lead); writing – original draft (supporting); writing – review and editing (equal).

## ACKNOWLEDGEMENTS

PS would like to thank the Ratanakiri Provincial Department of Environment, and in particular Thon Soukhon, for facilitating access to Virachey National Park, and Jeremy Holden for assisting with the deployment of camera traps. AT would like to thank Kathi Kasper and Thanh Nguyen for their help in providing second opinions on individual identification in the camera‐trap photos.

### OPEN RESEARCH BADGES

This article has earned an Open Data badge for making publicly available the digitally‐shareable data necessary to reproduce the reported results. The data is available at the following link: https://doi.org/10.5061/dryad.j3tx95xk3.

## Data Availability

The data that support this finding are archived in Dryad at the following link: https://doi.org/10.5061/dryad.j3tx95xk3.
